# Illuminating Messengers: An Update and Outlook on RNA Visualization in Bacteria

**DOI:** 10.3389/fmicb.2017.01161

**Published:** 2017-06-22

**Authors:** Lieke A. van Gijtenbeek, Jan Kok

**Affiliations:** Department of Molecular Genetics, Faculty of Science and Engineering, University of GroningenGroningen, Netherlands

**Keywords:** RNA visualization, fluorescence microscopy, aptamers, *in situ* hybridization, live cell imaging, bacteriological techniques

## Abstract

To be able to visualize the abundance and spatiotemporal features of RNAs in bacterial cells would permit obtaining a pivotal understanding of many mechanisms underlying bacterial cell biology. The first methods that allowed observing single mRNA molecules in individual cells were introduced by Bertrand et al. (1998) and Femino et al. (1998). Since then, a plethora of techniques to image RNA molecules with the aid of fluorescence microscopy has emerged. Many of these approaches are useful for the large eukaryotic cells but their adaptation to study RNA, specifically mRNA molecules, in bacterial cells progressed relatively slow. Here, an overview will be given of fluorescent techniques that can be used to reveal specific RNA molecules inside fixed and living single bacterial cells. It includes a critical evaluation of their caveats as well as potential solutions.

## Introduction

The rapid development and implementation of fluorescence microscopy techniques has led to the realization that, even in tiny bacterial cells, key molecular processes can be highly orchestrated and organized to temporarily or statically occur at specific subcellular sites (Rudner and Losick, 2010; Amster-Choder, 2011; Campos and Jacobs-Wagner, 2013). Most macromolecules, such as lipids, ribosomes, proteins, plasmids, metabolites, and RNA species move in varying diffusive states with different velocities in a crowded environment (Mika and Poolman, 2011). Therefore, the improvement of old and the advancement of new fluorescence-based tools to study molecular mechanisms and concurring macromolecules, while maintaining their spatial and temporal context, in individual bacterial cells are of prime importance to further expand our understanding of bacterial cell biology. This is also true for techniques to visualize and study events resulting from transcriptional and post-transcriptional regulation, which is ideally achieved by following single RNA molecules over time in living cells.

Initially, gene expression, like many other cellular processes, could only be studied in cultures of cells and, thus, in an ensemble fashion. In the latter approach, total RNA is usually extracted from tissue samples or cell cultures, after which it is further analyzed by Northern blotting, quantitative reverse transcriptase (qRT)-PCR, DNA microarrays or RNA-sequencing. This type of data yields important information but typically represents averaged and normalized values that cannot be regarded as quantitative or absolute. In addition, ensemble RNA measurements do not provide information on cell-to-cell variations in RNA content and intracellular spatiotemporal distributions of transcripts. Biological processes such as transcription are inherently stochastic and can show great differences within clonal populations, such as bacterial cultures (Norman et al., 2015). Tissues comprised of different cell types show even greater variations in gene expression; this information is averaged out when pooling RNA from multiple cells.

Fluorescent reporter protein fusions have also been employed to monitor the transcriptional activity of various promoters and to study the timing and level of gene expression in individual bacterial cells. In this way, the appearance of stochasticity and bistability in monoclonal bacterial populations of gene promoters as well as stress responses or changes in transcriptional activity could be elucidated at the single-cell level (For instance: Rosenfeld, 2005; Veening et al., 2008; Locke and Elowitz, 2009; Locke et al., 2011; Solopova et al., 2014). Although such fluorescent reporters are valuable tools for monitoring the activity of bacterial gene expression, they generate indirect and delayed information since the reporter proteins have to be translated, folded and fully matured before becoming fluorescent (Endoh et al., 2008). Moreover, the number of expressed genes that can be studied simultaneously is limited and fluctuations in expression within individual cells are hard to measure due to the stability of fluorescent proteins. Recently, next-generation sequencing has allowed transcriptome profiling at the single-cell level. Methods for single-cell RNA-seq in bacteria are under development. As of yet, these are technically very challenging because of the minute amounts of certain transcripts in single bacterial cells (<0.5 copies per cell in *Escherichia coli*) and therefore require a potentially bias-introducing pre-amplification step to prepare cDNA libraries (Taniguchi et al., 2010; Saliba et al., 2014; Liu and Trapnell, 2016).

Over the last decade, diverse methods have been developed to fluorescently label RNA molecules in individual cells. Besides providing insights into the subcellular quantity of certain RNA species and thus of transcriptional responses and population heterogeneity thereof, these methods also allow tracking the spatiotemporal attributes and, thus, the ultimate fate of these molecules. Specifically, *in vivo* RNA-labeling and -imaging tools alread*y* allow following the movement of transcripts inside eukaryotic cells over time and capturing transcription and even translation of mRNA *in vivo* and at the single-cell level (Katz et al., 2016; Morisaki et al., 2016; Wang C. et al., 2016; Wu et al., 2016; Yan et al., 2016). A variety of RNA-labeling methods similar to the ones employed in eukaryotic cells have been assessed in bacterial cells with a variety of outcomes as their applicability in bacterial cells has remained rather challenging (Golding and Cox, 2004; Golding et al., 2005; Valencia-Burton et al., 2009; Montero Llopis et al., 2010; Nevo-Dinur et al., 2011; dos Santos et al., 2012; Muthukrishnan et al., 2012; Makela et al., 2013; Zhang et al., 2015; Moffitt et al., 2016; van Gijtenbeek et al., 2016; Wang Y. et al., 2016). This is because only certain RNA-labeling methods are sensitive enough to detect native bacterial transcripts, many of which are characterized by transient lifetimes and low abundances. Despite their potential complications, methods that allow for studying RNA molecules in single cells represent a major advancement to study gene expression and the subsequent post-transcriptional status of these RNAs. For instance and importantly, different mRNA transcripts have been found to adopt distinctive subcellular sites in bacterial cells (Montero Llopis et al., 2010; Broude, 2011; Nevo-Dinur et al., 2011; dos Santos et al., 2012; Moffitt et al., 2016) and promoter parameters have been analyzed in great detail (For instance: Kandhavelu et al., 2011; So et al., 2011; Makela et al., 2013; Iyer et al., 2016; Sepulveda et al., 2016) using RNA-labeling techniques.

In what follows, we will provide a comprehensive review and discuss the current status of techniques that are used to image mRNA molecules in individual bacterial cells using fluorescence microscopy. Several approaches to tag RNA are available. These are based on three specific interactions of RNA with other macromolecules: Aptamer-protein interactions, aptamer-fluorophore interactions, and nucleic acid-probe annealing. We will elaborate on advances and pros and cons of the various methods, detailing recent developments for *in vivo* studies on mRNAs in eukaryotic cells and their feasibility for use in bacterial cells.

### RNA Visualization Techniques Based on Aptamer-Protein Interaction

**FIGURE 1:**
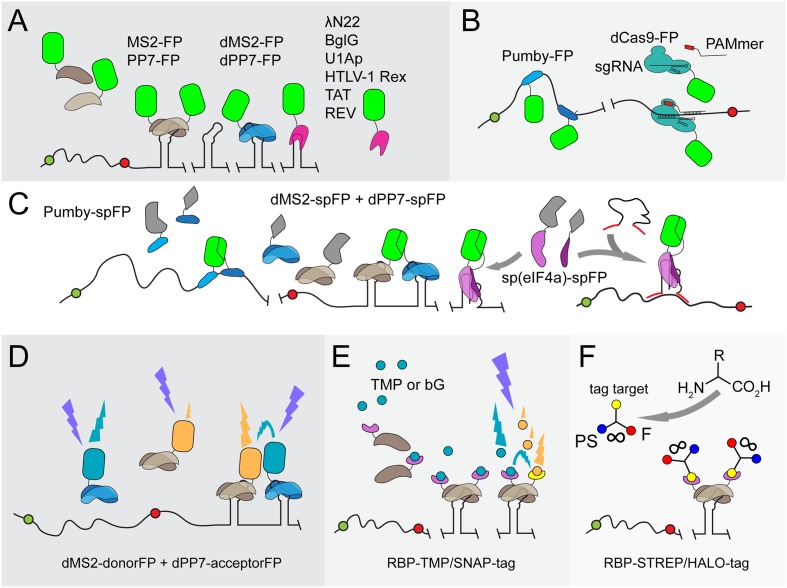
Overview of protein-aptamer-based mRNA visualization technologies. **(A)** First-generation protein-aptamer combinations. Aptamer-binding proteins are fused to a fluorescent protein (FP), such as GFP or YFP (green squircle), and expressed in the cell. The mRNA is extended with an array of aptamers. This array can either be introduced in the 5′ UTR before the start codon (green dot) or, as depicted here, behind the stop codon (red dot) and before the transcriptional terminator. Some aptamer binding proteins (MS2: brown shapes; PP7: blue shapes) bind to their cognate RNA aptamer as a dimer (Bertrand et al., 1998; Larson et al., 2011). Tandem repeats of these proteins have been constructed (dMS2 and dPP7) to improve binding kinetics (Wu et al., 2012). Alternative aptamer binding proteins (pink shape) are outlined in the right corner (U1Ap: Takizawa and Vale, 2000; λN22: Daigle and Ellenberg, 2007; BglG: Chen et al., 2009; HTLV-1 Rex: Yiu et al., 2011; TAT/REV: Yin et al., 2013). The major drawbacks of first-generation protein-aptamer systems are the substantial background fluorescence caused by unbound FPs and the need to fuse a long RNA array to the native mRNA, which might affect mRNA dynamics and degradation. **(B)** Protein-aptamer systems that target native transcripts. The modularity of Pumby subunits allows building proteins that recognize a specific sequence of ribonucleotides; multiple Pumby-FP variants (blue-shaded shapes with green squircle) can be expressed at the same time that concertedly bind to different positions on the same mRNA molecule (Ozawa et al., 2007; Adamala et al., 2016). The restriction-deficient Cas9 (dCas9) protein (turquoise shape) can be guided to specific RNA sequences by a sgRNA if a short DNA-based oligonucleotide (denoted as PAMmer) containing the PAM domain and a sequence complementary to target RNA (red box) is provided in *trans* (Nelles et al., 2016). **(C)** BiFC. To reduce background signals, the FP is split into two complementary parts (spFP; depicted as gray parts of a squircle). Each part can be fused to one of two RBPs that recognize two adjacent RNA sequence stretches on a native transcript (two Pumby proteins; blue-shaded shapes) or two adjacent aptamers (dMS2 as brown shapes and dPP7 as blue shapes) (Ozawa et al., 2007; Wu et al., 2014; Adamala et al., 2016). Alternatively, the RBP can be split into two parts that will complement each other upon binding to the original aptamer [such as split eIF4a, denoted as sp(eIF4a) and depicted as purple shapes] (Valencia-Burton et al., 2007). To achieve the detection of endogenous transcripts, the eIF4a-aptamer was extended with sequences complementary to the target RNA (red lines) (Toran et al., 2014). Upon hybridization, the aptamer will adopt its functional fold, triggering the reconstitution of the sp(eIF4a)-FP module. **(D)** FRET. Instead of employing a split FP, two FPs that constitute a FRET pair (orange and cyan squircles) can be attached to RBPs (blue and brown shapes). Careful control experiments are required to confirm the actual FRET signal. In addition, energy is lost during the transfer, leading to putatively weak fluorescence signals. dFP, donor FP; aFP, acceptor FP. **(E)** Fluorophore-binding peptide tags. An RBP-like MS2 (brown shape) is fused to a small peptide tag, such as the TMP-tag (purple extension) or SNAP-tag (yellow extension), that recognize a membrane-permeable fluorescent analog of their original target molecule TMP (trimethoprim; cyan dots) or bG (bG; orange dots), respectively (Carrocci and Hoskins, 2014; Xu et al., 2016). The level of fluorescent target molecules can be carefully controlled and stringent washing removes excess of unbound fluorophores. Two proximal peptide tags can be used to capture FRET-compatible fluorophores. **(F)** UAA scaffolds. Unnatural amino acids (UAAs) can be used as scaffolds to chemically link three chemical units (van der Velde et al., 2016). In this way, a photostabilizer (PS; blue dot) can be conjugated next to a synthetic organic fluorophore (F; red dot) to improve the photophysical properties of the latter i.a., reducing photobleaching and blinking of F (indicated by the infinity sign). Although not yet introduced to detect mRNA in living cells, an UAA conjugate of PS with F and for instance a SNAP-tag or HALO-tag target molecule (yellow dot) as a third moiety can interact with mRNA-bound RBP-SNAP-tag or RBP-HALO-tag fusions (brown shapes with purple extensions) in a cell, enabling prolonged mRNA imaging.

A multitude of RNA-binding proteins (RBPs) recognizes distinct RNA sequences with high affinity. One of the first high-specificity RNA-RBP pairs that was characterized originates from the *E. coli* MS2 phage (Peabody, 1990; Peabody and Ely, 1992; Ni et al., 1995; Peabody and Lim, 1996). The MS2 phage coat protein (designated MCP or, here, MS2) constitutes the icosahedral coat of the phage particle and also gathers the virus RNA in the virus particle (Ni et al., 1995). Repression of translation initiation of the viral replicase gene occurs when MS2 binds, as a dimer, to a short hairpin loop located on the virus RNA (Peabody, 1990; Peabody and Ely, 1992; Ni et al., 1995). Realizing that this protein-RNA interaction could be used as a basis to tag other RNA species, Singer and co-workers fused MS2 to GFP (Bertrand et al., 1998). By introducing multiple repeats of the RNA hairpin structure recognized by MS2 in the 3′-UTR of the yeast *ASH1* transcript they, and others, managed to visualize the hybrid RNA in living yeast cells (Bertrand et al., 1998; Beach et al., 1999). These studies paved the way for a plethora of follow-up experiments. The MS2 system is used in yeast cells to follow single mRNA molecules in space and time and is also employed in plant, bacterial, and mammalian cells such as neurons (Rook et al., 2000; Fusco et al., 2003; Zhang and Simon, 2003; Golding and Cox, 2004). More recently, it has been adapted for transcript visualization in living transgenic zebrafish and developing *Drosophila* embryos (Forrest and Gavis, 2003; Campbell et al., 2015). The MS2 system can be combined with analogous systems, such as PP7 coat protein (designated PCP or, here, PP7) and lambda N protein in concert with their cognate aptamers, to study the spatiotemporal behavior of multiple transcripts, at the same time, in living cells (Lange et al., 2008; Hocine et al., 2013). In 2016, at least four groups introduced various techniques to track single mRNAs and translation thereof *in vivo* by simultaneously visualizing fluorescently tagged transcripts and their nascent peptides (Katz et al., 2016; Morisaki et al., 2016; Wang C. et al., 2016; Wu et al., 2016; Yan et al., 2016). A summary of all RBP-aptamer pairs that have been used for the visualization of RNA molecules is given in **Figure 1**.

#### MS2-Tagging in a Bacterial Perspective

Although protein-based RNA labeling is now widely employed in eukaryotic organisms, several caveats have thwarted the extensive use of MS2-based RNA labeling and similar systems in bacteria. The main problem is that most employ intact autofluorescent proteins, causing substantial background noise that obscures the proper visualization of single transcripts. The background noise is effectively reduced in eukaryotic cells by tagging unbound MS2-GFP proteins with a nuclear localization signal (NLS) sequence, which directs the surplus of NLS-MS2-GFP proteins to the nucleus (Bertrand et al., 1998). Such compartmentalization does not exist in bacteria and other approaches have therefore been explored to increase the signal-to-noise ratio in bacterial cells.

Golding and Cox were the first to successfully transfer the MS2 system to living *E. coli* cells to study transcription kinetics (Golding and Cox, 2004; Golding et al., 2005). The use of an array consisting of 96 MS2 binding sites (*ms2*) transcriptionally fused to the transcript under study resulted in sufficient fluorescent signal to visualize and follow single mRNA molecules over time in individual *E. coli* cells. In comparison, the incorporation of 24 binding sites typically suffices to monitor single transcripts in eukaryotic cells. It was recognized that extensive tethering of MS2-GFP reduces the diffusion rate of the transcript under study and renders the molecule perpetual (Golding and Cox, 2004; Golding et al., 2005; Montero Llopis et al., 2010; Garcia and Parker, 2015). Golding and colleagues employed this transcript immortality to monitor transcription since it results in an additive increase of fluorescence signal that can be used to calculate the number of mRNA molecules produced over a set period of time (Golding et al., 2005; So et al., 2011). Evidently, MS2-GFP-based mRNA labeling is not suitable to study highly dynamic, short-lived transcripts that are only present in small numbers. However, when MS2-GFP is expressed to the correct level with respect to available *ms2* sites, the technique can be used to identify variations in mRNA localization patterns within a range of bacterial species (Nevo-Dinur et al., 2011; dos Santos et al., 2012; van Gijtenbeek et al., 2016).

#### Recognition of Native Transcripts

The mRNA molecule has to be genetically modified in most protein-based RNA-labeling systems in order to introduce multiple repeats of the RNA ligand (**Figure 1A**). This can be a laborious task and, as discussed above, may influence the endogenous properties of the transcript under study. The RNA-labeling system employing PumHD and homologous proteins does not suffer from the latter drawback (**Figure 1B**; Ozawa et al., 2007). PumHD belongs to the eukaryotic protein family of conserved PUF proteins that modulate gene expression through binding to the 3′ UTR of target mRNAs (Quenault et al., 2011). The PUF protein RNA-binding domains consist of eight very similar motifs that each recognizes one specific RNA base. This modularity allows modifying the recognition code: only two amino acids in a domain have to be altered in order to establish recognition of another ribonucleotide (Wang et al., 2002). A PUF protein with 16 RNA binding repeats further increases the binding specificity (Filipovska et al., 2011). More recently, Pumilio-based assembly (Pumby) modules have been developed that allow researchers to generate specific protein-RNA interactions (Adamala et al., 2016). Four Pumby building blocks were selected that each recognizes one of the four RNA bases. These blocks can be concatenated to bind virtually any ribonucleotide sequence without the need to modify or extend the endogenous mRNA sequence. The number of binding blocks in the protein can be varied to achieve increased selectivity. Interestingly, Pumby proteins are displaced from transcripts by ribosomes, resulting in a loss and reconstitution of the signal, which can be used to monitor translation (Adamala et al., 2016). Due to their modularity, Pumby blocks are suitable for multiplexing and can be designed to bind UTRs or small non-coding RNAs. Taking these features together, Pumby blocks show great promise for future studies involving RNA tagging, also in bacteria, although one has to keep in mind that protein mutagenesis of Pumby modules might be a time-consuming undertaking.

The RNA-guided Cas9 protein of the bacterial CRISPR-Cas9 immune system is a DNA endonuclease that was long thought to solely recognize DNA targets. The site where Cas9 interaction with DNA occurs is dictated by the combination of the protospacer adjacent motif (PAM) domain in the target DNA and the Cas9-bound single-guide (sg) RNA. Recently, Cas9 was found to also recognize single-stranded RNA after slight modifications of the CRISPR/Cas9 system (O’Connell et al., 2014): The sgRNA guides Cas9 to a target RNA if a short DNA-based oligonucleotide that contains the PAM domain and a sequence complementary to target RNA (denoted as PAMmer) are both provided *in trans*. Non-tagged endogenous mRNAs were isolated from HeLa cells using this principle (O’Connell et al., 2014). It was suggested that CRISPR-Cas9 could also serve as a generic tool for *in vivo* RNA visualization (Nelles et al., 2015). As a proof of principle, mammalian cells were transfected with a plasmid expressing nuclease-inactive Cas9-GFP and a specific sgRNA and provided with a synthetic nuclease-resistant PAMmer (Nelles et al., 2016). Subsequently, endogenous mRNA molecules could be accurately targeted and visualized (**Figure 1B**). It remains to be elucidated whether this system is functional in bacteria to track individual transcripts due to a potentially insufficient signal amplification.

#### Improving Signal-to-Noise Ratios of RBP Systems

To reduce signal-to-noise ratios, care needs to be taken in expressing the proper number of RBP-GFP molecules in relation to the estimated number of available RNA target binding sites. Even low expression of MS2-GFP may lead to a surplus of unbound MS2-GFP proteins in the cytoplasm, which would thwart capturing single mRNA molecules using fluorescence microscopy. This is true for all RBP-FPs expressed in bacterial cells in which RBP-FPs cannot be contained within a specific compartment. Even though Pumby and Cas9 have the advantage of targeting endogenous mRNAs, the background noise issue remains. It is essential to carefully consider which RBP is the right choice for RNA visualization in bacteria. For instance, a detailed analysis of the binding kinetics of MS2 and PP7 to their respective aptamers revealed that PP7 binds with a higher affinity than MS2 because PP7 occupies more aptamer repeats. Importantly, the construction of a tandem dimer of both MS2 and PP7 increased protein-RNA interactions in various eukaryotic cell lines, effectively improving signal-to-noise ratios (Wu et al., 2012). Bacterial RNA tagging may benefit from these adjustments even though improved binding kinetics would never fully eliminate (high) background fluorescence. Consequently, new adjustments and techniques have been developed that incorporate molecular tools such as protein complementation, Förster resonance energy transfer or aptamers that bind fluorogenic dyes. These approaches will be discussed in the following sections.

#### Protein Complementation (PC)

Protein complementation employs split fluorescent proteins that reconstitute into a functional fluorescing protein upon reunion via the interaction of their respective fusion partners (Shyu and Hu, 2008). The implementation of PC has been explored in RNA-labeling systems to reduce background fluorescence. For instance, Broude and colleagues developed a bacterial RNA-labeling system based on split domains of eukaryotic initiation factor-4A (eIF4a; **Figure 1C**; Valencia-Burton et al., 2007, 2009; Yiu et al., 2011). Later on, eIF4a-based RNA labeling was ingeniously modified to detect single endogenous mRNA molecules in bacteria (**Figure 1C**; Toran et al., 2014). In this approach, the split RNA aptamer is extended, at both sites, with a sequence complementary to the target RNA and is produced, like the two split eIF4a-GFP parts, inside the bacterial cell using an expression vector. Upon head-to-tail hybridization of the complementary sequence extensions of the RNA modules with the target RNA, the aptamer reassembles, yielding a binding site to reunite the bifurcated eIF4a-GFP protein parts. In another study, MS2 and PP7 were each fused to one half of a split YFP while the RNA of interest was appended with a chimeric aptamer repeat array consisting of alternating MS2 and PP7 binding sites, abrogating background fluorescence (**Figure 1C**; Wu et al., 2014). Also, the Pumilio-based systems described above have been successfully adapted to a tetra-molecular fluorescence complementation (TetFC) approach in which one half of a split GFP was fused to one PUM 8-mer recognizing a specific RNA sequence and the other half to a second PUM 8-mer that binds to an adjacent RNA sequence (**Figure 1C**; Wang et al., 2002; Ozawa et al., 2007; Adamala et al., 2016).

Although RNA-labeling techniques based on split proteins reduce background fluorescence to almost zero, several issues have prevented the further development of this method for RNA tagging in bacteria. First, reassembly of split fragments is mostly irreversible as only a small fraction of reconstituted fluorescent proteins will disassemble into split parts again. The reduction of background fluorescence will therefore not be as high as expected. Second, the split fluorescent protein domains can interact with each other without the need for association of their fusion partner - the aptamer-binding protein -, which necessitates the inclusion of many control experiments. Third, like all fluorescent proteins split fluorescent proteins require time to fully mature and become functional. The maturation process includes protein folding and a torsional rearrangement of the fluorophore (the active site of the protein), followed by cyclization and a final oxidation step in order for the active site to fluoresce. In addition to folding, the last step can take a relatively long time and greatly depends on the presence of sufficient molecular oxygen. Maturation of full-length super-folder GFP is estimated to take around 11 min under optimal conditions (Fisher and DeLisa, 2008). A commonly used split-GFP, split-sg100-GFP, has a much lower maturation rate. It takes between 24 and 72 h after reassembly before reconstituted fluorescence is detected (Magliery et al., 2005). Unlike eukaryotic mRNAs that typically have relatively long lifetimes, most mRNAs in *E. coli* are degraded within 3–10 min post-synthesis (Bernstein et al., 2002). Thus, the maturation time of split fluorescent proteins greatly exceeds the timing of most mRNA-related processes in bacteria. Recent improvements in split-GFP maturation have yielded split folding-reporter-GFP (split-frGFP) and split super-positive GFP (split-spGFP) (Sarkar and Magliery, 2008; Blakeley et al., 2012). Both show an increase in fluorescence development and reassembly rates compared to split-sg100-GFP in *E. coli* grown at 37°C. Reconstituted fluorescence was observed within 1 h, which makes these split-GFP variants more suitable for live cell approaches in general. Nonetheless, the kinetics of reconstitution and dissociation of split fluorescent proteins variants are not yet good enough to capture the spatiotemporal parameters of the mostly short-lived mRNA species in bacteria.

#### Förster Resonance Energy Transfer (FRET)

Förster Resonance Energy Transfer fluorescence microscopy makes use of a donor fluorophore that dissipates energy to and thereby excites a nearby (<10 nm) acceptor fluorophore upon excitation. Donor-acceptor pairs can exist of fluorophores and/or fluorescent proteins. Live single-cell and single-molecule FRET studies have been executed and well-performing FRET pairs consisting of fluorescent proteins have been carefully selected for various bacterial species (Alexeeva et al., 2010; Detert Oude Weme et al., 2015; van der Ploeg et al., 2015; Bajar et al., 2016). A combination of MS2-based RNA tagging and FRET has been proposed (Dictenberg, 2012) but only a few studies have adopted a FRET-based strategy to reduce signal-to-noise ratios in live protein-based RNA tagging (**Figure 1D**). Rather, RNA binding mediated FRET (RB-FRET) was developed to detect the interaction between eukaryotic RBPs and their cognate RNA (Rackham and Brown, 2004; Lorenz, 2008; Huranova et al., 2009). FRET is considered a valuable technique but requires extensive optimization, normalization and control experiments. Weak fluorescence and difficulties in data interpretation have further frustrated the development of FRET in protein-based mRNA tagging.

#### Peptide Tags

The introduction of fluorophore-binding peptides to image RNA might offer a solution to the high background fluorescence evoked by unbound autofluorescent aptamer-binding fusion proteins (**Figure 1E**). The MS2 protein can be fused to, for instance, a trimethoprim (TMP)-tag (dihydrofolate reductase from *E. coli*; eDHFR) or a SNAP-tag (a 20-kDa mutant of the human DNA repair protein O6-alkylguanine-DNA alkyltransferase) (Carrocci and Hoskins, 2014; Xu et al., 2016). The TMP-tag and the SNAP-tag interact with fluorescent analogs of trimethoprim (TMP) and benzyl guanine (bG), respectively. Since these fluorophores are membrane permeable they can diffuse into and out of cells. Straightforward washing steps will therefore suffice to eliminate unbound fluorophores from the cellular environment, thereby efficiently increasing signal-to-noise ratios. It has to be noted, however, that TMP is a known broad-spectrum antibiotic. Therefore, the usability of the TMP-tag in bacteria relies on the concentration of TMP required to obtain a detectable signal. A multitude of fluorophore-binding peptide tags is available to achieve single-step protein visualization, including the HALO-tag, the CLIP-tag, the STREP-tag, the BirA-tag, the ReacTR-tag and the Tris-NTA tag [for an excellent review, see (Yan and Bruchez, 2015)]. The STREP-, SNAP- and HALO-tags have been successfully employed in *E. coli* and *Salmonella enterica* as the respective small-molecule ligands readily diffuse through both bacterial membranes (Landgraf et al., 2012; Barlag et al., 2016; Ke et al., 2016). Many membrane-permeable fluorophores can be combined for multiplexing or the generation of FRET-pairs.

Although the genetically encoded self-labeling protein tags can be relatively bulky and may require longer incubation times depending on the tag and cell type, they allow detecting mRNAs in live cells with photostable fluorophores. The latter is key to achieving single-molecule tracking and super-resolution microscopy (Dempsey et al., 2011). Fluorophores can be further stabilized with ‘healing’ buffers or via the attachment of photostabilizers. Recently, a generic method has been developed to generate conjugates of synthetic organic fluorophores with photostabilizers target molecules using unnatural amino acids (UAAs) as a scaffold (van der Velde et al., 2016). The use of photostabilizers improves the photophysical properties in terms of photostability and sensitivity of various dyes. Three functional groups can be attached to a UAA scaffold. Hence, a third molecular group can be attached to the scaffold next to the fluorophore and the photostabilizer. Interestingly, most ligands of the self-labeling protein tags described above are compatible with UAA scaffolding, which, apart from single-molecule examination, might enable long-term tracking of mRNA molecules (**Figure 1F**). Hence, self-labeling protein tags hold great promise for both the visualization of RNA molecules and proteins, also because the diversity of commercially available tags and fluorophores is rapidly growing.

### RNA Labeling by Small-Molecule Binding Aptamers

**FIGURE 2:**
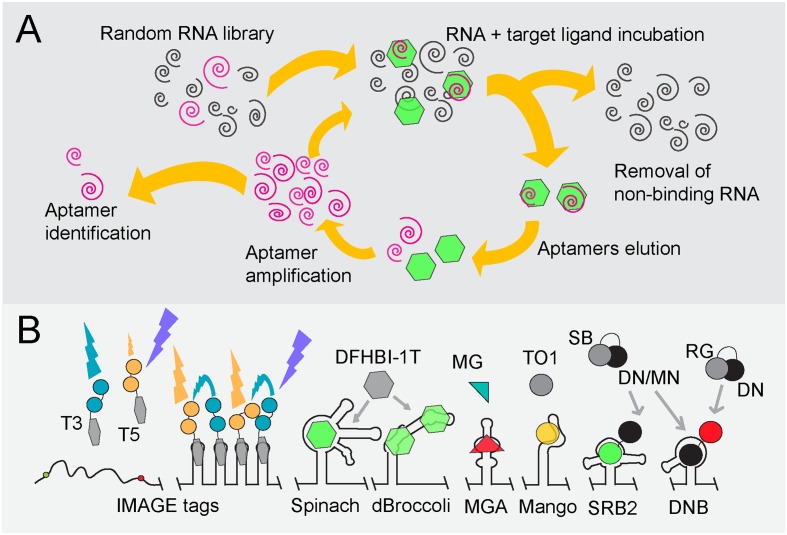
Overview of fluorogen-binding aptamers for single-cell mRNA detection. **(A)** SELEX (Stoltenburg et al., 2007). Schematic representation, starting at the top-left, of SELEX (Systematic Evolution of Ligands by Exponential Enrichment) to identify RNA aptamers of high affinity (pink spirals) to target ligands (green hexagons). **(B)** Fluorogen, fluorophore and quencher-binding aptamers. An array of tobramycin-binding RNA aptamers (IMAGE tags) guide the generation of FRET when both tobramycin-Cy3 (T3) and tobramycin-Cy5 (T5) are fed to cells in a 1:1 mixture (Shin et al., 2014). Binding of the non-fluorescent molecule DFHBI-1T (gray hexagon) by Spinach or Broccoli derivatives (dBroccoli) forces DFHBI-1T to adopt its fluorescent conformation (green hexagon) (Paige et al., 2011; Filonov et al., 2014; Song et al., 2014). The same principle is used for the Malachite Green (green/red triangle)-binding aptamer (MGA; Babendure et al., 2003) and the RNA MANGO aptamer that interacts with acetylated thiazole orange (TO1; gray/yellow dot; Dolgosheina et al., 2014). Quencher-fluorophore conjugates such as sulforhodamine B-dinitroaniline (SB-DN), sulforhodamine B-p-nitrobenzyl-amine (SB-MN) or Rhodamine green-dinitroaniline (RG-DN) are non-fluorescent in solution (depicted as gray and black cherry-like structure). Displacement of the quencher away from the fluorophore, either by the interaction of the SB fluorophore with the SB binding (SRB-2) aptamer or of the DN quencher binding (DNB) aptamer, respectively, restores fluorescence of SG (green dot) or RG (red dot) (Sunbul and Jäschke, 2013; Arora et al., 2015).

As mentioned above, certain small organic dyes are ideal candidates for RNA-labeling techniques because they can diffuse through cell membranes. SELEX (Systematic Evolution of Ligands by Exponential Enrichment) is a combinatorial method for the generation of nucleic acids binding to virtually any molecule, including proteins and small molecules such as organic dyes and metabolites (**Figure 2A**; Stoltenburg et al., 2007). The implementation of SELEX has greatly appended the number of available aptamers, next to those already naturally present. SELEX has yielded nucleic acids that bind small organic dyes such as sulforhodamine B (SB), malachite green (MG), fluorescein (FL), rosamine (RM) and 5-carboxytetramethylrhodamine (TAMRA) at high specificity and affinity *in vitro* (Holeman et al., 1998; Grate and Wilson, 1999; Babendure et al., 2003; Carothers et al., 2010). However, *in vivo* applications have remained scarce for most of the developed RNA-dye pairs due to limitations in fluorescence shifts or cellular toxicity. To detect and visualize an RNA species *in vivo*, an aptamer is required that significantly changes, activates or dramatically enhances the fluorescence of the selected dye upon binding to the aptamer. This minimizes intracellular background fluorescence, which enables the detection and quantification of tagged RNA molecules. Employing RNA-dye interactions eliminates the need for the attachment of bulky proteins to visualize RNA, thereby providing a non-intrusive way to image RNA.

#### FRET

As discussed, FRET can circumvent non-specific background signals caused by unbound proteins or other aptamer-ligands. Intracellular MultiAptamer GEnetic tags (IMAGE tags) have been designed to visualize RNA through fluorophore-based FRET (**Figure 2B**; Shin et al., 2014). Transcripts can be extended with an array of tobramycin-binding aptamers, after which both tobramycin-Cy3 and tobramycin-Cy5 are fed to cells in a 1:1 mixture. The alternating binding of tobramycin-Cy3 and tobramycin-Cy5 to the aptamer array creates a FRET signal that is distinguishable from that of unbound probes. Unfortunately, like TMP, tobramycin is a toxic compound that functions as an antibiotic, especially for Gram-negative bacteria.

#### Conditionally Fluorescent (Fluorogenic) Dyes

Fluorogens are small-molecule dyes that show no or little fluorescence in solution but become fluorescent by binding to aptamers or peptides [reviewed in Hori and Kikuchi (2013)]. To date, only a few fluorogen-aptamer pairs have been reported to be functional in a cellular environment (**Figure 2B**), of which the Spinach RNA-fluorophore pair was reported first (Paige et al., 2011). Spinach is a 98-nt-long RNA sequence that was evolved to bind 3,5-difluoro-4-hydroxybenzylidene imidazolinone (DFHBI), a compound that resembles the fluorogenic core of GFP. Unbound DFHBI dissipates its energy through intramolecular motions, rendering it non-fluorescent in free solution. Interaction with the Spinach aptamer reduces these motions, yielding fluorescence upon excitation (excitation/emission: 469/501 nm). Spinach and its derivatives have found many applications due to the low background signals and the cell-permeating ability of DFHBI. Spinach has also been adapted to sense metabolites such as cyclic d-AMP and to follow transcriptional events (Höfer et al., 2013; Kellenberger et al., 2013; Song et al., 2013; Pothoulakis et al., 2014; Wang X.C. et al., 2016). In addition, a split-Spinach variant was developed that forms a promising tool for monitoring RNA processing (Rogers et al., 2015; Ausländer et al., 2016).

Initially, Spinach suffered from impaired folding and thermal instability of the relatively long RNA structure, hampering the fluorescence development of DFHBI in a cellular environment (Strack et al., 2013). The presence of a tRNA scaffold flanking the aptamer improves the folding stability of Spinach (Paige et al., 2011). In addition, Spinach-fluorogen complexes undergo photoconversion and fast fluorescence decay upon illumination, which was later on found to be reduced by pulsed illumination (Han et al., 2013). Further adaptations led to the development of the Spinach2 and Broccoli RNA aptamers (**Figure 2B**). Spinach2 shows improved folding kinetics through the presence of stabilizing RNA hairpins but still requires a tRNA scaffold for optimal functionality (Strack et al., 2013; Strack and Jaffrey, 2015). This is the core drawback of Spinach and derivatives in general: every adjacent RNA region may greatly influence folding of the aptamer. Even though the stabilizing tRNA scaffold counteracts this problem, tRNA is targeted by bacterial and mammalian ribonucleases, effectively reducing Spinach levels (Filonov et al., 2015). Hence, fluorescent signals obtained with the Spinach variants are not sufficient to reveal cellular RNA levels (Shin et al., 2014). A major improvement in DFHBI-based RNA visualization was obtained by replacing the final SELEX screening step by selecting RNA aptamers that display bright fluorescence of RNA in *E. coli* using fluorescence-activated cell sorting (FACS). In this way, the 49-nt RNA molecule Broccoli was developed (**Figure 2B**; Filonov et al., 2014). As Broccoli was selected for improved folding kinetics and fluorescence in a cytoplasmic environment, it is dramatically less dependent on the magnesium concentration. In addition, the tRNA scaffold was replaced by F30, a nuclease-resistant and stabilizing RNA element that further improves Broccoli fluorescence in living cells. Tandem Dimeric Broccoli (tdBroccoli) as well as some Broccoli derivatives with stabilized quadruplex-flanking stem structures display enhanced brightness and hold great promise for *in vivo* RNA expression studies (Ageely et al., 2016; Filonov and Jaffrey, 2016).

Spinach and Broccoli have not allowed visualizing low-abundance RNAs. Since most RNA species are present in low numbers (<0.5 mRNA molecules per *E. coli* cell), it would be highly desirable to be able to detect these molecules through RNA labeling. A recent study shows that arranging multiple Spinach aptamers in a tandem array increases fluorescence (Zhang et al., 2015). It was estimated that each doubling of Spinach aptamers increases fluorescence by 1.6-fold. However, high numbers of Spinach repeats also lead to a reduced folding efficiency of the aptamers. Repetitive laser pulse illumination allows visualizing low-copy-number mRNAs using Spinach tandems in *E. coli* (Zhang et al., 2015). Tandem arrays of Spinach2 and the malachite green aptamer (discussed below) have been developed, each of which shows improved fluorescence-signal-to-noise ratios (Ilgu et al., 2016)

Malachite green (MG) is a triphenylmethane dye with low quantum yield in solution. The fact that the autofluorescence of MG increases in cold or viscous environments led Babendure and colleagues to develop a 38-nt-long MG-binding aptamer (MGA) that resulted in an over 2000-fold increase and red-shift in MG fluorescence (**Figure 2B**; Babendure et al., 2003). It was used to detect half-lives of RNA nanoparticles following their introduction in cells (Reif et al., 2012). Recently, MGA was employed to detect RNA in CHO cells but imaging of the MG-MGA-tagged RNA was only possible for up to 10 min, after which an accumulation of background fluorescence by non-specific interactions of MG with other intracellular molecules prevented further RNA imaging (Ilgu et al., 2016).

The recently introduced Mango RNA aptamer (**Figure 2B**) binds derivatives of thiazole orange (TO) at high binding affinities in the presence of potassium (Dolgosheina et al., 2014). Capturing acetylated and biotinylated TO (TO1-Biotin) by RNA Mango resulted in a 1,100-fold increase in fluorescence (excitation/emission: 510/535 nm) compared to uncomplexed TO1-Biotin. RNA Mango in combination with TO1-biotin was shown to be applicable in live *Caenorhabditis elegans* syncytial gonads and single *E. coli* cells. Interestingly, RNA Mango can be integrated into RNA structures that carry non-functional loops due to its structural composition, as exemplified by the ability to monitor *E. coli* 6S RNA in an RNA-Mango dependent fashion (Dolgosheina et al., 2014).

In addition to refining aptamer properties, fluorogenic dyes have been improved for fluorescence microscopy through photophysical modifications. A modified DFHBI version, DFHBI-1T, carries a trifluoro-ethyl substituent and displays lower background fluorescence as well as a slight red shift in its excitation spectrum [excitation/emission: 482/505 nm; (Song et al., 2014)]. It is, therefore, better suited than DFHBI for imaging of both Spinach and Broccoli derivatives using standard fluorescence microscopy equipment. PFP-DFHBI, a DFHBI derivative with a 5-Pentafluorophenyl (PFP) group, has increased affinity for Spinach and an improved fluorescence yield (Ilgu et al., 2016). TO3-Biotin, a derivative of TO, was synthesized as a proof-of-principle to demonstrate the ease at which the spectral properties of TO can be chemically changed. TO3-Biotin has a red-shifted spectrum compared to that of TO and has an excitation peak at 637 nm in complex with RNA Mango (Dolgosheina et al., 2014).

#### Fluorophore-Quencher Conjugates

A quencher is a molecule that effectively reduces fluorescence emitted by a fluorophore whenever both are in close proximity. They can be conjugated to fluorophores such that no fluorescence is emitted when the probe is free in solution. Quenching can progress through various mechanisms. Of these, static (or contact) quenching is most effective. Static quenching is completely abolished when the quencher-fluorophore complex becomes dismantled (Arora et al., 2015). Quenchers have been used in a delicate way to achieve background-free aptamer-based fluorescence. For instance, binding of the fluorophore-quencher couple Sulforhodamine B-dinitroaniline (SB-DN) to the sulforhodamine-binding RNA (SRB-2) aptamer leads to the displacement of the DN quencher away from the SB fluorophore (**Figure 2B**), resulting in a 100-fold increase in SB fluorescence (Sunbul and Jäschke, 2013). SRB-2-tagged RNA can be clearly visualized with SB-DN in individual *E. coli* cells, indicating that both the folding stability of the SRB-2 aptamer and binding of SB-DN are compatible with live imaging of bacterial cells.

Contact quenchers such as DN block fluorescence of a wide variety of fluorophores. Therefore, instead of designing an aptamer that binds existing fluorogens, a DN-binding (DNB) aptamer was introduced (Arora et al., 2015). Binding of DNB to a DN-fluorogen conjugate leads to a structural rearrangement of the DN-fluorogen pair and to fluorescence (**Figure 2B**). Importantly, non-conditional fluorophores with different spectral properties, such as rhodamine green, tetramethylrhodamine or sulforhodamine can be used in combination with the same DNB aptamer (Arora et al., 2015). To this end, the DN quencher conjugated to SB was replaced by the alternative quencher p-nitrobenzylamine, through which SRB-2 and DNB aptamers can be simultaneously used in *E. coli* cells (**Figure 2B**; Arora et al., 2015).

Future studies will have to show if recent improvements of existing aptamers are applicable and truly robust enough for *in vivo* imaging of RNAs in bacterial cells or whether further advances of current or new aptamers are needed to obtain systems that accurately function in an intracellular environment.

### RNA Tagging Using Nucleic Acid-Probe Annealing Techniques

The oldest technique to visualize RNA in individual cells using fluorescence microscopy is based on *in situ* hybridizations of oligonucleotide probes to complementary nucleic acid sequences (Singer and Ward, 1982). Although aptamer-based techniques as described in the previous sections have gained much attention, fluorescent *in situ* hybridization (FISH) has remained the predominant tool for the examination of cell-to-cell variations in transcript numbers. The technique itself was initially developed to visualize DNA but adapted to allow quantification and localization of mRNA in single cells using fluorescence microscopy. Contemporary RNA FISH protocols use oligonucleotides of 17–22 nucleotides that are complementary to the RNA under study (Raj and van Oudenaarden, 2009; Skinner et al., 2013). The oligonucleotides are typically pre-labeled with a fluorochrome or another reactive moiety. Fluorophore-based mRNA tagging is extensively used and has improved considerably over the past years. A set of straightforward FISH protocols now enables scientists to detect mRNA molecules at the single-molecule level. Continuous developments have led to many variations on the original technique, such as multiplexed error-robust FISH (MERFISH), which allows detecting up to thousands of different RNA species simultaneously (Chen et al., 2015; Moffitt and Zhuang, 2016), fluorescence *in vivo* hybridization (FIVH) for live-cell mRNA imaging in mammalian cells (discussed below) and Turbo RNA FISH in which the hybridization time is increased by changing to alcohol-based fixation and increasing probe concentration (Shaffer et al., 2013).

#### Fluorescent *In Situ* Hybridization (FISH)

**FIGURE 3:**
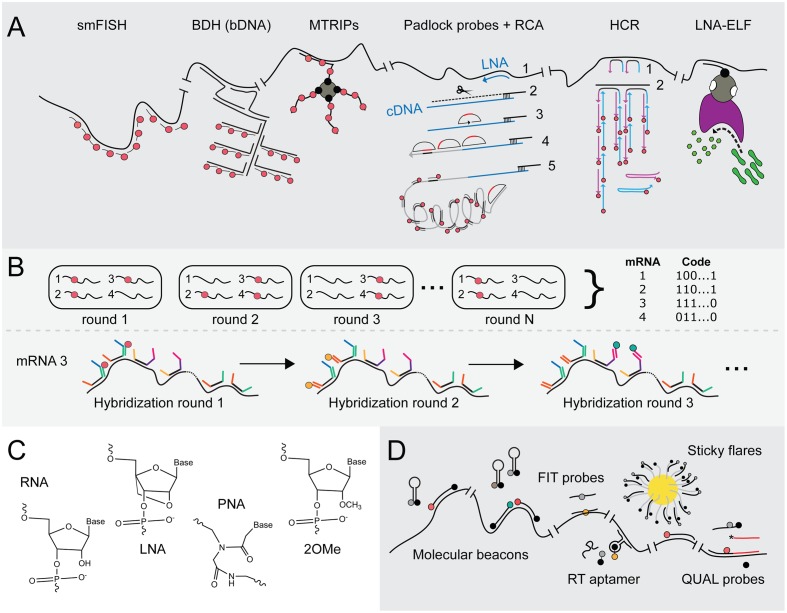
Advances in FISH and FIVH methods for the visualization of mRNAs at single-molecule resolution. **(A)** Signal amplification techniques to achieve smFISH. smFISH (Raj and van Oudenaarden, 2009): The most common method to achieve smFISH is to design multiple probes (red tadpoles) that each hybridizes to a 20-nt sequence of the target transcript. BDH (Player et al., 2001): Branched DNA (bDNA) hybridization (BDH) can be employed to maintain probe-annealing specificity. In the first hybridization step, two Z-shaped capture probes recognize the target transcript. These probes jointly constitute an annealing site for the capture extender oligonucleotide (L shapes). The latter contains hybridization sites for fluorescent probes (red tadpoles). The capture probes are the only sequence-specific subunits in this system and, thus, are the only probes that require redesign if another transcript has to be targeted, whereas the capture extender and the fluorescent probes are universal. MTRIPs (Santangelo et al., 2009): multiply-labeled tetravalent RNA imaging probes consist of a tetravalent streptavidin molecule (gray circle) to which four biotin-labeled fluorescent probes (red dots) are attached via their biotin-moieties (black dots). The recognition of one probe arm is sufficient to elevate the fluorescent signal above background fluorescence. Padlock probes + RCA (Larsson et al., 2004, 2010): Rolling circle amplification using a single padlock probe: A highly specific LNA probe (blue arrow) is used for a reverse transcriptase reaction (1), creating an mRNA-DNA hybrid. The mRNA is then partially degraded using RNase H (black scissors; 2). A padlock probe (half-moon shape) hybridizes to the cDNA (blue line) that is still anchored to the non-degraded part of the mRNA molecule (3). cDNA that stretches beyond the padlock probe is degraded, while the knick formed by the two ends of padlock probe is ligated, yielding a circular probe that serves as a primer during RCA (4). One fluorescently labeled probe (red tadpole) can now be hybridized to repeats (black stripes) within the rolling circle product (gray line), creating a significant and spatial-conserved signal. (5) HCR (Choi et al., 2010, 2014): Hybridization chain reaction. DNA initiator probes hybridize with the target sequence (1), after which toeholds on the probes’ 3′ and 5′ end (blue and purple extensions) can bind fluorescent probes (blue and purple arrows), thereby opening the metastable hairpins in the latter (2). A new initiator segment is generated that can function as a new toehold for hybridization with a second probe, resulting in a chain reaction of probe hybridization at the mRNA target and signal amplification. (6) LNA-ELF-FISH (Lu and Tsourkas, 2009): A biotin-labeled LNA probe (black tadpole) hybridizes to the mRNA target sequence. A streptavidin-alkaline phosphatase (AP) conjugate (gray-pink shape) binds to the biotin moiety (black dot on tadpole). To visualize mRNA, enzyme-labeled fluorescence (ELF) signal amplification is performed by feeding AP (pink shape) with 2-(5′-chloro-2-phosphoryloxyphenyl)-6-chloro-4(3H)-quinazolinone (green peanuts). After cleavage by AP, a yellow-green fluorescent precipitate in the proximity of AP activity is formed. **(B)** MERFISH (Chen et al., 2015) employs multiple rounds of probe hybridization to visualize many transcripts at the same time. Via a sequential labeling scheme, the identity of each targeted transcript can be decoded. In the first annealing step, multiple probes bind to all the mRNA targets. These initial probes consist of a highly specific sequence complementary to that of its target and two or three additional sequences that form targets for fluorescently labeled probes. In the first hybridization round, a fluorescent probe (red tadpole) is added with one specific sequence, which therefore binds to transcripts that contain an initial probe carrying the complementary sequence. These are then washed or cleaved off, after which the second fluorescent probe (orange tadpole) is added that binds to the second target sequence. In this way, transcripts do or do not sequester fluorescent probes during subsequent hybridization round, leading to a specific binary code which allows deducing the identity of many transcripts in the same cell. **(C)** Molecular structures of RNA, LNA, PNA and 2′-O-methylated (2OMe) backbone units. RNA: ribose moieties. DNA: deoxyribose backbone (lacking 2′-hydroxyl group). LNA (Obika et al., 1997): modified RNA nucleotide in which the 2′ oxygen and 4′ carbon are connected, such that the ribose is in a locked conformation. PNA (Nielsen et al., 1991): N-(2-aminoethyl)-glycine units that are connected via peptide bonds and therefore electrically neutral. 2OMe-ribonucleotides (Majlessi et al., 1998): the ribose moiety contains a 2′-methoxy group instead of a 2′-hydroxyl group, which leads to faster hybridization and higher melting temperatures. **(D)** FIVH technologies. Molecular beacons (Tyagi and Kramer, 1996): The 5′ and 3′ ends of molecular beacons are labeled with a quencher and a fluorophore (black and gray dots in cherry-like shape). An unbound beacon undergoes intermolecular base pairing by which the fluorophore and quencher stay in close proximity. The molecular beacon opens upon annealing to target RNA, rendering fluorescence (red dot). Two molecular beacons can be employed to create a FRET pair (red and green dots). FIT probes (Köhler et al., 2005): Intercalation probes that contain a fluorogenic base (gray dot) which is forced to adopt its fluorescent conformation (yellow dot) upon hybridization to target RNA. RT aptamer (Sato et al., 2015): The core domain of the RNA-targeting (RT) aptamer consists of an unstable aptamer that binds the black hole quencher (BHQ; black part of dumbbell), whereas the outer regions contain complementary sequences to the target RNA. The RT aptamer only adopts a conformation that stably binds BHQ and the target RNA when the latter molecules are both present. BHQ loses its quenching capacity upon stable probe binding, yielding fluorescence of the fluorophore (orange dot) conjugated to BHQ. Sticky flares (Briley et al., 2015): Gold nanoparticles (yellow circle) conjugated to oligonucleotides (tadpoles with gray tails and black heads). These oligonucleotides function as docking sites for fluorescent hybridization probes (tadpoles with black tails and gray heads). The fluorescence of probes is quenched as long as they remain bound to the gold nanoparticle, but will fluoresce (depicted by red tadpole) once they detach and bind the target RNA instead. QUAL probes (Sando et al., 2004): Two 2OMe-based probes are introduced in the cell that anneal head-to-tail to the target RNA. The first probe contains a fluorophore (gray/red dot) and a dabsyl quencher (black dot). The second probe contains a reactive phosphorothioate group (asterix) that attacks and thereby removes the dabsyl moiety when the probes hybridize next to each other.

FISH was one of the first techniques facilitating the *in situ* visualization of single molecules (smFISH) in individual cells (Femino et al., 1998). Initially, FISH experiments suffered from high background fluorescence caused by unspecific and unbound oligonucleotide probes, obscuring the distinction between true and false-positive signals. Also, the incorporation of fluorophores and the hybridization efficiency hampered effective detection of the mRNAs under study. Signal-to-noise ratios were too low to detect single mRNA molecules, but these problems were solved in several ways. The hybridization of target transcripts with many singly labeled short oligonucleotides results in sufficient specificity and amplifies the signal above background (Raj and van Oudenaarden, 2009). Alternatively, transcripts can be appended with a repetitive sequence to which one probe can bind multiple times although this requires genetic modification of the target sequence (**Figure 3A**). Both strategies have been successfully applied to quantify endogenous transcripts in *E. coli* (So et al., 2011; Skinner et al., 2013; Fei et al., 2015; Arbel-Goren et al., 2016; Moffitt et al., 2016; Sepulveda et al., 2016) and an extensive protocol is available for the quantification of gene expression in bacteria by means of smFISH (Skinner et al., 2013). Using different sets of fluorescent probes that either tag the head, the body, or the tail of a specific transcript, smFISH can also be employed to reveal events such as transcription initiation, elongation and transcript degradation (Iyer et al., 2016).

Scaling up of probe numbers and/or hybridization sites effectively relieve the methods’ shortcoming of yielding great variations in signals due to non-optimal probe hybridization and labeling. However, this adjustment does not work in case short RNA species such as bacterial small RNAs have to be visualized using fluorescence microscopy techniques. Surrogate signal amplification can be achieved through several innovative strategies, including the use of branched DNA (bDNA) hybridization (BDH) (Player et al., 2001), multiply-labeled tetravalent RNA imaging probes (MTRIPS) (Santangelo et al., 2009), Padlock probes followed by rolling circle amplification (RCA) (Larsson et al., 2004, 2010), hybridization chain reaction (HCR) (Choi et al., 2010, 2014) and enzyme-labeled fluorescence (ELF)-based signal amplification (Paragas et al., 1997; Lu and Tsourkas, 2009) see **Figure 3A** for and overview; excellent reviews on these innovations can be found in Pitchiaya et al. (2014) and Gaspar and Ephrussi (2015).

Backbone modification of oligonucleotide probes has further increased binding specificities (**Figure 3C**). Locked nucleic acid (LNA) probes are more rigid because the 2′ oxygen and the 4′ carbon in the RNA nucleotide are linked, leading to highly specific Watson-Crick base pairing (Obika et al., 1997; Koshkin et al., 1998). Peptide nucleic acid (PNA) probes consist of an uncharged peptide backbone (Nielsen et al., 1991; Egholm et al., 1993). The lack of negatively charged phosphate groups creates an increase in PNA probe-RNA binding compared to DNA probe-RNA binding and, thus, in enhanced specificity (Egholm et al., 1993; Socher et al., 2008).

Multiplexed error-robust FISH was introduced by the group of Zhuang in 2015 and is a strategy based on multiplexed fluorescence *in situ* hybridization with high-performance (Chen et al., 2015; Moffitt and Zhuang, 2016). Elaborated and updated protocols and analysis tools are available on the website of the research group^1^. It allows visualizing up to 140 mRNA molecules at the same time in single cells in an error-robust fashion. The technology helps in understanding how expression profiles vary between single cells and how different sets of transcripts are spatially organized at the subcellular level. MERFISH employs a two-step hybridization scheme in combination with combinatorial labeling through which each targeted mRNA sequence is translated to and can be identified via a unique binary code (**Figure 3B**). Importantly, this technique has been successfully applied in single *E. coli* cells, in which it has led to the acknowledgment that mRNAs that include genes encoding membrane proteins are generally localized in proximity of the cytoplasmic membrane, whereas mRNA species that only contain open reading frames for soluble, secretory or outer membrane proteins are scattered throughout the cytoplasm (Moffitt et al., 2016).

#### Fluorescence *In Vivo* Hybridization (FIVH)

In order to introduce multiple nucleic acid-based probes into a cell, chemicals are added that fixate the cell contents and permeabilize the cytoplasmic membrane. Fixation inactivates nucleases, thus preventing the rapid degradation of single-stranded DNA probes and RNA-DNA hybrids. Notwithstanding this, in an ideal situation mRNAs are studied in living bacterial cells. Fluorescence *in vivo* hybridization (FIVH) can be achieved but requires alternative probe delivery and circumvention of at least the degradation of probes by cellular nucleases. Synthetic probes, such as LNA and PNA probes, but also 2′-*O*-methylated oligonucleotides (2OMe; **Figure 3C**) display improved nuclease stability, which make them especially suited for *in vivo* imaging of RNA (Majlessi et al., 1998). In addition, a bright fluorescent signal has to be generated by probe-target interactions while background fluorescence by unbound probes has to be minimal.

Molecular beacons are modified FISH probes that consist of a binding domain, complementary to the target RNA, and two flanking regions that base pair when the probe is unbound (Tyagi and Kramer, 1996; Matsuo, 1998; Sokol et al., 1998; **Figure 3D**). One end of the probe contains an organic fluorophore, while the other is labeled with a quencher that keeps the fluorophore in a dark state as long as the termini of the probe are in close proximity. Quenching is alleviated when the molecular beacon adopts a linear structure upon interaction with target RNA, yielding fluorescence. mRNAs have been visualized *in vivo* via microinjection of 2OMe-based molecular beacons in a variety of mammalian cells (Tyagi and Kramer, 1996; Matsuo, 1998; Sokol et al., 1998; Bratu et al., 2003). It was soon realized that most probes that were either microinjected or introduced using pore-forming agents were in fact sequestered in the nucleus, obscuring mRNA visualization in the cytoplasm. This was overcome by linking a tRNA moiety to a molecular beacon since tRNAs mainly function in the cytoplasm and are exported from the nucleus to the cytoplasm (Mhlanga, 2005). Signal-to-noise ratios can be further enhanced by using two molecular beacons of which the two fluorophores form a FRET pair, albeit that this approach lowers the fluorescence quantum yield due to a significant loss of energy (Santangelo, 2004). The most dominant disadvantages of molecular beacons include the instability of the hairpin structure, for instance by non-specific interaction with other macromolecules, and the standard occurrence of some residual quenching after base pairing of the beacon to mRNA.

Light-Up, Forced intercalation (FIT) and Exciton-controlled hybridization-sensitive fluorescent oligonucleotide (ECHO) probes consist of PNA or 2OMe-based backbones that carry a fluorogenic base (Svanvik et al., 2000; Köhler et al., 2005; Kubota et al., 2009; Okamoto, 2011). The incorporated fluorogen thiazole orange (TO; see above) exhibits low fluorescence activity whenever the FIT or ECHO probe to which it is bound is free in solution. TO undergoes forced intercalation upon hybridization of the probe with target RNA, resulting in a significant increase in fluorescence (**Figure 3D**; Bethge et al., 2010). Both ECHO and FIT probes are suitable for *in vivo* mRNA imaging upon delivery into living eukaryotic cells (Kubota et al., 2009, 2010; Hövelmann et al., 2013, 2014; Okamoto et al., 2013). Interestingly, injection of approximately four FIT probes gives comparable signals as those obtained with the MS2 system in living *Drosophila* oocytes (Hövelmann et al., 2014).

The so-called black hole quencher (BHQ) aptamer initially showed insufficient functionality as mRNA appendix and was modified into a probe that anneals to target RNA (**Figure 3D**; Sato et al., 2015). Three base pairs in the stem-loop of the BHQ aptamer were maintained, while each end was extended with ribonucleotide strands complementary to the target transcript, resulting in the RNA targeting (RT) aptamer. The RNA-targeting strands interfere with the formation of a stable BHQ-binding conformation in the unbound probe but are at the same time dependent on BHQ interaction for their hybridization to target RNA. Thus, only in the presence of BHQ and target RNA does the probe form a stable BHQ-binding loop and bind to specific RNA. In this way, the corresponding BHQ–bound fluorophore becomes unquenched. Because the RT aptamer only hybridizes to mRNA in the presence of BHQ–fluorophore, the RT aptamer can be co-expressed in cells without affecting the endogenous target mRNA. This concept renders the system suitable for application in living cells. To further increase sensitivity, multiple RT aptamers, that target different sites on a native RNA, could be co-expressed in living cells.

Gold nanoparticles covered with DNA-based oligonucleotides hybridized to so-called “Nanoflares” or “Sticky flares” can be endocytosed by mammalian cells without the need for transfection. The second-generation Sticky flares, illustrated in **Figure 3D**, consist of a dye-conjugated DNA-based backbone complementary to both the oligonucleotide attached to the nanoparticles and the target RNA (Seferos et al., 2007; Prigodich et al., 2012; Briley et al., 2015). The fluorescence of the dye on the Sticky flares is quenched as long as the latter remain attached to the nanosphere, but will become detectable if the Sticky flares detache from the nanosphere and basepair with adjacent target RNA instead (Briley et al., 2015). Moreover, the short double-stranded DNA sequences on Sticky flares are not degraded by cellular nucleases. Since bacteria do not perform endocytosis, alternative methods to introduce flares should be explored to set up this technique in bacterial cells (see below).

Modified nuclease-resistant probes can be introduced in living eukaryotic cells by microinjection or via pore-forming peptides. Such strategies have not yet been published for bacterial cells. Hence, smFISH is traditionally performed on fixed permeabilised bacterial cells with DNA-based probes, resulting in mere snapshots of the processes under study. LNA and PNA probes are, however, widely employed for bacterial strain identification via 16S rRNA FISH because of their higher specificity over DNA oligonucleotides. Interestingly, quenched autoligating (QUAL) probes (**Figure 3D**) were used to detect 16S rRNA sequences in living *E. coli* cells with high specificity (Sando et al., 2004). The QUAL probe system is based on the use of a DNA probe pair of which one probe is unlabeled and contains a 3′-phosphorothioate group, whereas the other contains a quenching dimethylamino-azobenzenesulfonyl (dabsyl) group adjacent to a fluorophore at its 3′-end. The adjacent binding of the two probes on the target sequence leads to removal of the dabsyl moiety, which is converted by an attack of the phosphorothioate group on the unlabeled probe. Removal of the dabsyl quencher subsequently leads to fluorescence. The use of QUAL probes with a 2OMe-backbone results in slightly better signals but to a reduced specificity due to an increase in binding affinity (Silverman and Kool, 2005). Highly specific hybridization was achieved with 9-mer dabsyl DNA probes, enabling the discrimination between 16S rRNAs of three closely related and live bacteria, *E. coli*, *S. enterica*, and *Pseudomonas putida*. To deliver QUAL probes to the cytoplasm of live bacterial cells, the latter were incubated with probes in a hybridization buffer consisting of saline-sodium citrate (SSC) and sodium dodecyl sulfate (SDS) surfactant (Sando et al., 2004; Silverman and Kool, 2005). Both SSC and SDS affect the viability of cells in a dose-dependent fashion, but their addition to a specific concentration is required to obtain the desired signals (Franzini and Kool, 2011). Whether signals are bright enough for the detection of single molecules remains to be elucidated.

Of the developed FIVH techniques for use in mammalian cells, the RT aptamer has two major advantages. First, it is the only FIVH system that does not require physical introduction into the cells. Second, it offers a mechanism to limit background fluorescence. As discussed, the RT aptamer can be expressed in cells and is, therefore, very likely functional in bacteria. Although protection against ribonucleases may be limited, a continuous expression of RT aptamers abolishes the need for precarious probe delivery via microinjection or pore-forming compounds and therefore shows great potential for the establishment of similar techniques in bacterial cells.

### Enzymate-Based Strategies to Light Up RNA

Last but not least, efforts are undertaken to covalently label RNAs in cells with reporter molecules via so-called bio-orthogonal “click” chemistry, which often makes use of one-pot azide-alkyne cycloaddition (AAC) to achieve bioconjugation. The RNA of interest can be post-synthetically and chemo-enzymatically modified by transferases to contain click-compatible moieties that are then joined to other substrates through biocompatible AAC (reviewed in Phelps et al., 2012). Alternatively, azide- or alkyne-containing UTP analogs can be incorporated by RNA polymerases (Sawant et al., 2016). In both approaches, the major pitfall is that the incorporation of clickable groups does not provide substrate selectivity. tRNA-modifying enzymes are promising candidates in this respect because they modify one tRNA species with high precision, resulting in site-specific labeling. The tRNA^Ile2^-modifying enzymes present in prokaryotes, archaea and eukaryotes differ in that they recognize non-similar, unique sequence domains. The archaeal tRNA^Ile2^-agmatidine synthetase (Tias) can therefore be used in mammalian 293T cells to specifically incorporate an azide-carrying agmatine in 5S RNA carrying a genetically encoded archaeal tRNA^Ile2^-tail, after which the tRNA^Ile2^-5S fusion RNA can be fluorescently labeled with Sulfo-Cy5-azide via AAC (Li et al., 2015). In order to achieve the described Tias-based RNA labeling, both the archaeal tRNA^Ile2^-modifying and the cognate tRNA recognition site have to be absent, which is the case in mammalian cells. The most common click reaction involves CuI-catalyzed azide-alkyne cycloaddition (CuAAC), which requires the incorporation of toxic Cu(II) molecules. Therefore, the studies presented above utilized cell fixation prior to CuAAC. However, copper-free AAc reactions, like strain-promoted AAc (SPAAC), could be employed to realize site-specific and covalent RNA labeling in living cells.

## Outlook and Discussion

Studies that focus on the spatiotemporal behavior and abundance of diverse RNA molecules help to gain deeper insights into the real-time organization of genetic information in bacteria. In contrast to the progress made for analyses in eukaryotic cells, the development of *in vivo* RNA imaging techniques for individual bacterial cells has progressed more slowly. The biggest challenge in this area is to obtain sufficiently high signal-to-noise ratios without influencing the endogenous behavior of the RNA under study. Furthermore, the technique should ideally be compatible with acquiring high-frequency images or long-term time-lapse movies to investigate RNA dynamics. A comprehensive overview including the pros and cons of the RNA-labeling techniques discussed in this review is given in **Table 1**.

**Table 1:** Summary and review of methods to visualize RNA in single bacterial cells.

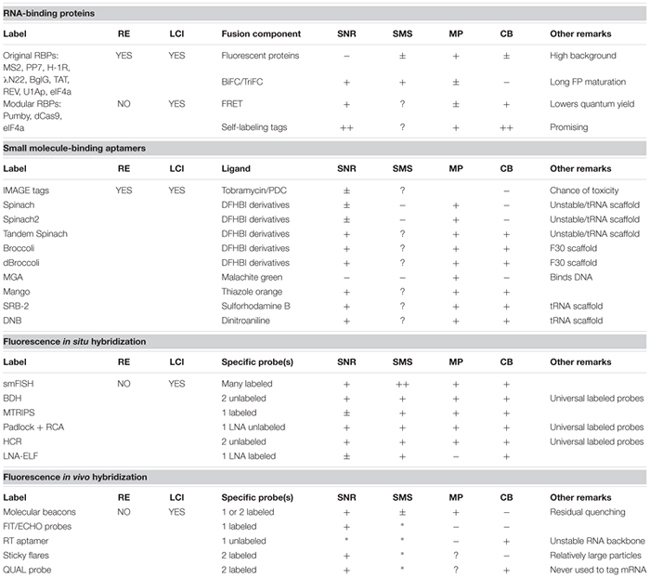

*For references and abbreviations of labeling systems, see main text and figure legends. Additional abbreviations: H-1R, HTLV-1 Rex; RE, RNA extension required; LCI, live cell imaging; SNR, signal-to-noise ratio; SMS, single molecule sensitivity; MP, multiplexing, imaging of multiple transcripts in the same cell; CB, compatible with bacterial cells. Scoring index: ++, very suitable; +, suitable;±, not ideal; -, not suitable; ?, unknown; ^∗^, likely possible with modifications.*

RNA-binding protein-based techniques, such as the MS2, PP7 and eIF4A systems have been adapted for use in bacterial cells more than a decade ago but have, unfortunately, met with limited success. Although MS2 can be used to follow transcript production in time, one has to keep in mind that the diffusion and localization of MS2-labeled transcripts are severely estranged from the natural situation due to the addition of bulky complexes. The number of MS2-binding appendices can be reduced when studying high-abundant RNAs and/or by careful fine-tuning of the ratio between target RNAs and RBP levels. However, this would still not allow quantifying low-abundant or single RNA molecules to reflect native cell genetics. New RBP-based methods have recently emerged that make use of Cas9 or Pumby modules (Adamala et al., 2016; Nelles et al., 2016). Their use circumvents the need to extend target RNA, but rather allows labeling of the endogenous transcripts. Localization via Pumby proteins, Cas9 or other RBPs is typically achieved using fluorescent proteins. Notwithstanding this, the value of fluorescent proteins in RNA, and for that matter, in protein-labeling techniques is slowly diminishing. This is not only because of the relatively bulky size of fluorescent proteins but also because organic fluorophores display significantly better photophysics in terms of stability and brightness than fluorescent proteins. Multiple Pumby modules in combination with the proper fluorophore-binding peptide tags have a true potential in boosting *in vivo* RNA labeling in bacteria and would even allow single-molecule visualization and tracking. Also, the recently developed RNA-targeting (RT) aptamer is a bright light in bacterial mRNA detection as the system combines the advantages of targeting native transcripts with high specificity and bright and stable fluorophores.

To date, smFISH is the only available technique to reliably count the number of mRNA molecules in bacterial cells while at the same time acquiring data with high spatial resolution (Skinner et al., 2013; Fei et al., 2015; Moffitt et al., 2016; Sepulveda et al., 2016). Recent improvements in signal-to-noise ratios and binding specificity through backbone modification of the probes now make it possible to detect shorter RNA fragments, such as eukaryotic microRNAs or bacterial small non-coding RNAs (Hernandez et al., 2013; Fei et al., 2015; Arbel-Goren et al., 2016). Furthermore, multiplexing strategies have been developed that incorporate spectral barcoding or an iterative hybridization/imaging procedure (MERFISH), in which probes with different fluorophores that sequentially target mRNAs are designed in such a way that careful analysis of the color combination within one mRNA spot informs on the identity of that transcript (Levsky et al., 2002; Lubeck and Cai, 2012; Jakt et al., 2013; Chen et al., 2015; Moffitt and Zhuang, 2016). In this way up to thousands of RNAs can be detected in one single cell. Thus, smFISH is extremely useful for monitoring gene expression and transcript localization. One major drawback remains, however, namely that FISH is not compatible with *in vivo* imaging of bacteria. Rather, it conveys static data of - in fact – highly dynamic processes. On top of that, cell fixation required for FISH could also influence the localization of the studied RNA by itself.

Novel modifications deal with most shortcomings of FISH in live eukaryotic cells. Alterations that turn FISH into FIVH include backbone changes that render probes resistant to cellular nucleases and a variety of signal amplification strategies, such as the development of molecular beacons and dye-intercalating probes. However, delivery of these relatively large oligonucleotides into the cell requires microinjection, transfection or the action of pore-forming peptides. These techniques are not (yet) compatible with the small-sized and cell wall-enclosed bacteria. Electroporation can be used to internalize short DNA fragments or proteins up to 100 kDa in *E. coli* while at the same time keeping cells in a viable state (Plochowietz et al., 2014; Sustarsic et al., 2014; Aigrain et al., 2015; Di Paolo et al., 2016). It would be of great interest to elucidate whether fluorescently labeled FIVH probes, such as molecular beacons or LIT probes, could be introduced in bacteria in a similar fashion. Probes are sometimes introduced in eukaryotic cells using cell-penetrating peptides like streptolysin O. Along this line of thought, pore-forming compounds such as the antibiotic peptide nisin may be used to temporarily permeabilize Gram-positive membranes (Wiedemann et al., 2001). More recently, advances in microfluidic cell “squeezing” resulted in the formation of short-lived holes in the membranes of mammalian cells, allowing introducing small synthetic fluorophores without affecting cell viability. The feasibility of this technique for the relatively rigid bacterial cells remains to be examined (Kollmannsperger et al., 2016). Alternatively, living cells can be incubated in hybridization buffer with low concentrations of SSC and SDS with the knowledge that this will negatively affect probe hybridization or cell viability. Possibly, probe introduction and hybridization in stringent hybridization buffer, followed by careful washing with a less stringent buffer, could improve survival.

The shortfall of FISH in imaging RNAs in living bacterial cells has led to the development of methodologically different RNA-labeling techniques. Approaches that have received the most attention in recent years make use of aptamer-binding fluorogenic dyes. Many small organic dyes are able to diffuse into cells and are good candidates for less intrusive RNA imaging strategies. A number of fluorogen-aptamer pairs have been reported for live-cell RNA imaging, of which most required considerable optimization after their first introduction. Next-generation aptamers are now coming of age. Additional technical improvements and development of new fluorogen-binding aptamers circumvent many of the problems that are encountered with protein-based RNA-labeling techniques and, thus, hold great promise for future *in vivo* RNA studies. Moreover, the recently developed aptamers and their organic dye ligands are, in theory, compatible with other aptamer-dye pairs by taking advantage of the variation in spectral characteristics of the fluorophores. Especially those techniques that would allow detecting single RNA molecules for prolonged periods of time would boost the knowledge on bacterial cell biology. So far, all fluorogen-binding aptamer methods that have been used to detect transcripts in bacteria have employed multicopy plasmids to express the mRNA. Also, all of the studies have so far been undertaken in *E. coli* only. These are clear indications of the fact that most techniques are still under development and none have yet yielded physiological information on endogenous bacterial transcripts, many of which are typically low-abundant and short-lived.

## Conclusion

Although monitoring of single mRNA molecules in bacteria has proven difficult, important information has been gained regarding the spatial behavior of RNA species inside bacterial cells. The techniques have allowed uncovering the transcription kinetics of promoter systems in *E. coli* at the single-cell level (Kandhavelu et al., 2011; So et al., 2011; Lloyd-Price et al., 2012; Muthukrishnan et al., 2012; Iyer et al., 2016; Sepulveda et al., 2016) and led to the acknowledgment that various mRNAs are in fact localized in different ways in bacterial cells (Nevo-Dinur et al., 2011; dos Santos et al., 2012; Moffitt et al., 2016). The systems that were used to enable these pioneering studies are now gradually being replaced by more accurate, less invasive techniques. Importantly, many resolutions to initial drawbacks in the procedures have been proposed but remain to be validated for their use in bacterial cells. It is therefore very likely that a generic RNA-labeling system will be presented in the near future, allowing to make major steps in the understanding of the hidden and long-underestimated subcellular localization and dynamic behavior of bacterial RNA molecules.

## Author Contributions

LvG has drafted the manuscript. JK and LvG have reviewed and edited the manuscript.

## Conflict of Interest Statement

The authors declare that the research was conducted in the absence of any commercial or financial relationships that could be construed as a potential conflict of interest.

## Footnotes

1 http://zhuang.harvard.edu/merfish/
